# Supplementary figures and images for: Unraveling the unbinding pathways of SARS-CoV-2 Papain-like proteinase known inhibitors by Supervised Molecular Dynamics simulation
Source: PLoS One. 2021 May 19;16(5):e0251910. doi: 10.1371/journal.pone.0251910 (PMC8133426; doi:10.1371/journal.pone.0251910)

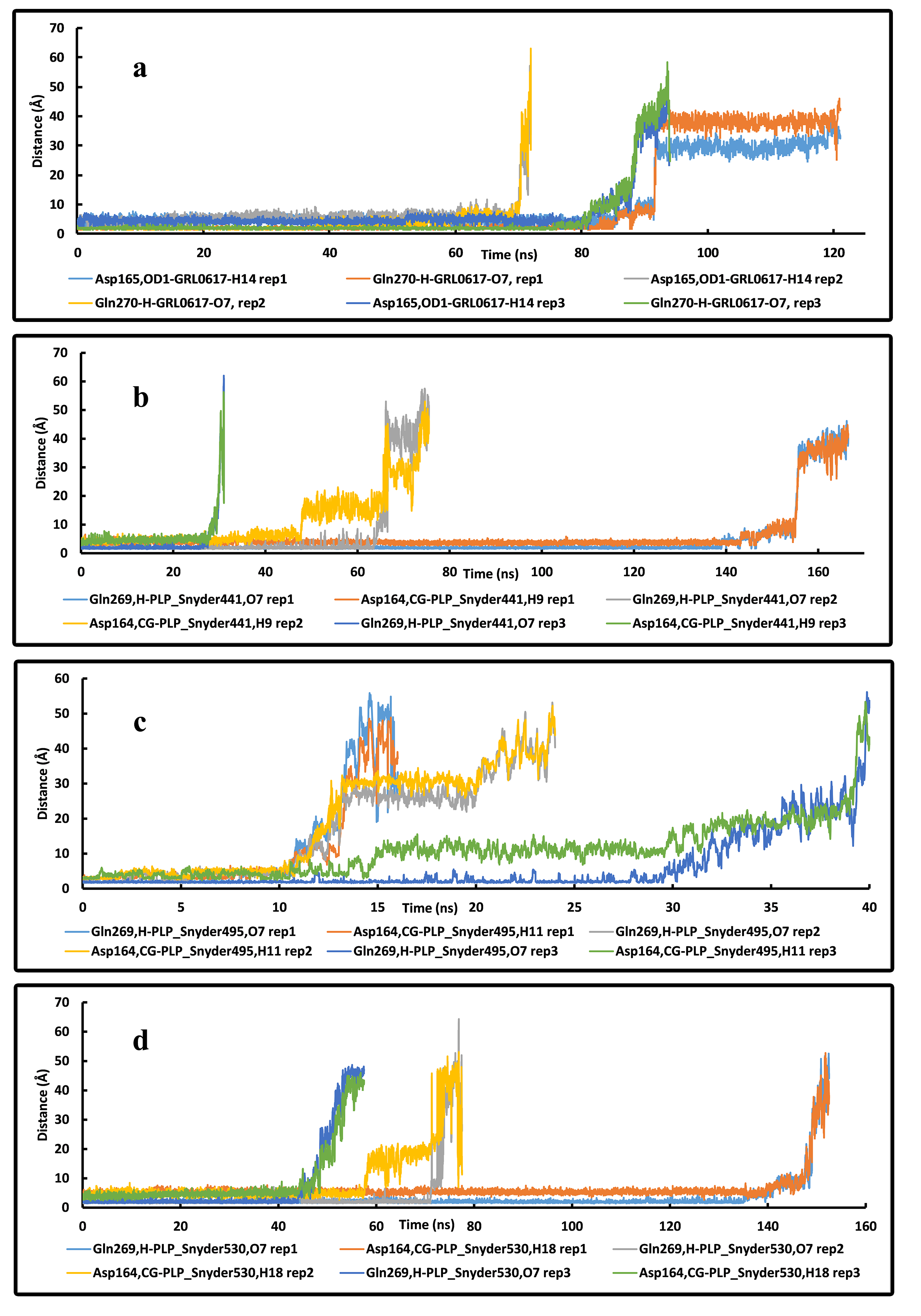

Supplement: S1 Fig — (A) The important hydrogen bonds in the GRL0617-Plpro complex. (B) The important hydrogen bonds in PLP-Snyder441-Plpro complex. (C) The important hydrogen bonds in PLP_Snsyder495-Plpro complex. (D) The important hydrogen bonds in PLP_Snsyder530-Plpro complex. (TIF) [file pone.0251910.s001.tif]

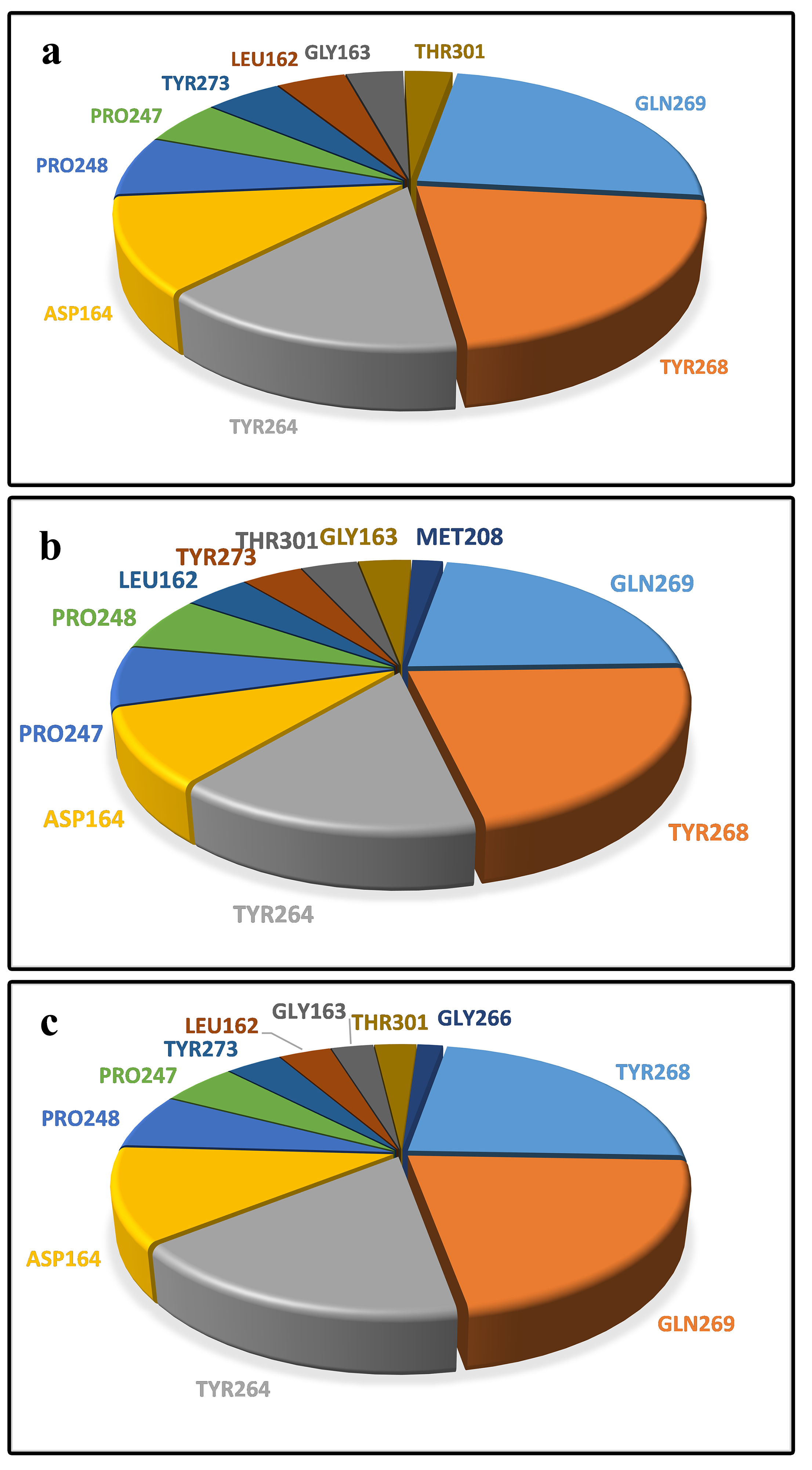

Supplement: S2 Fig — A) replica No 1. (B) replica No 2. (C) replica No 3. (TIF) [file pone.0251910.s002.tif]

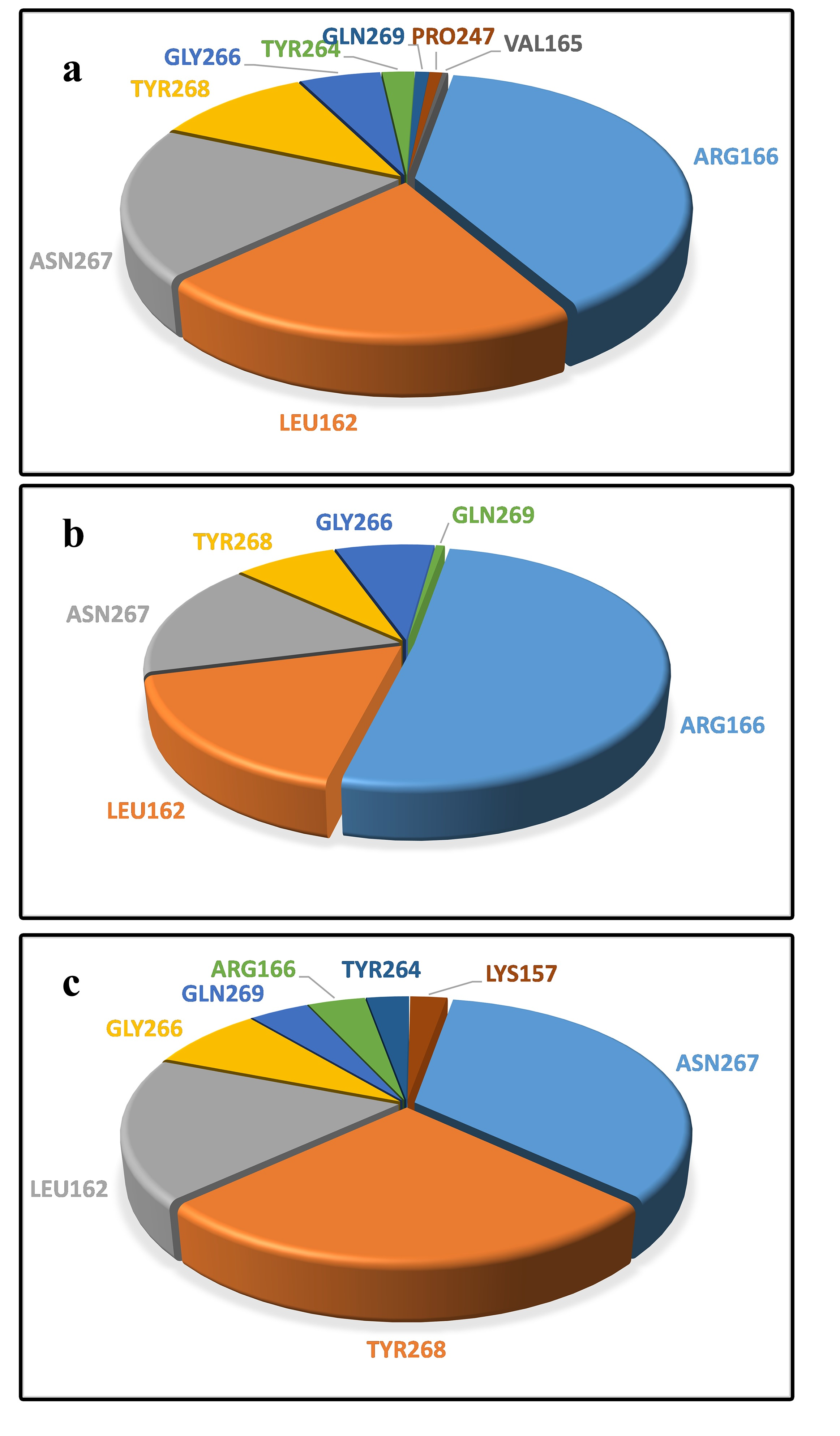

Supplement: S3 Fig — (A) replica No 1. (B) replica No 2. (C) replica No 3. (TIF) [file pone.0251910.s003.tif]

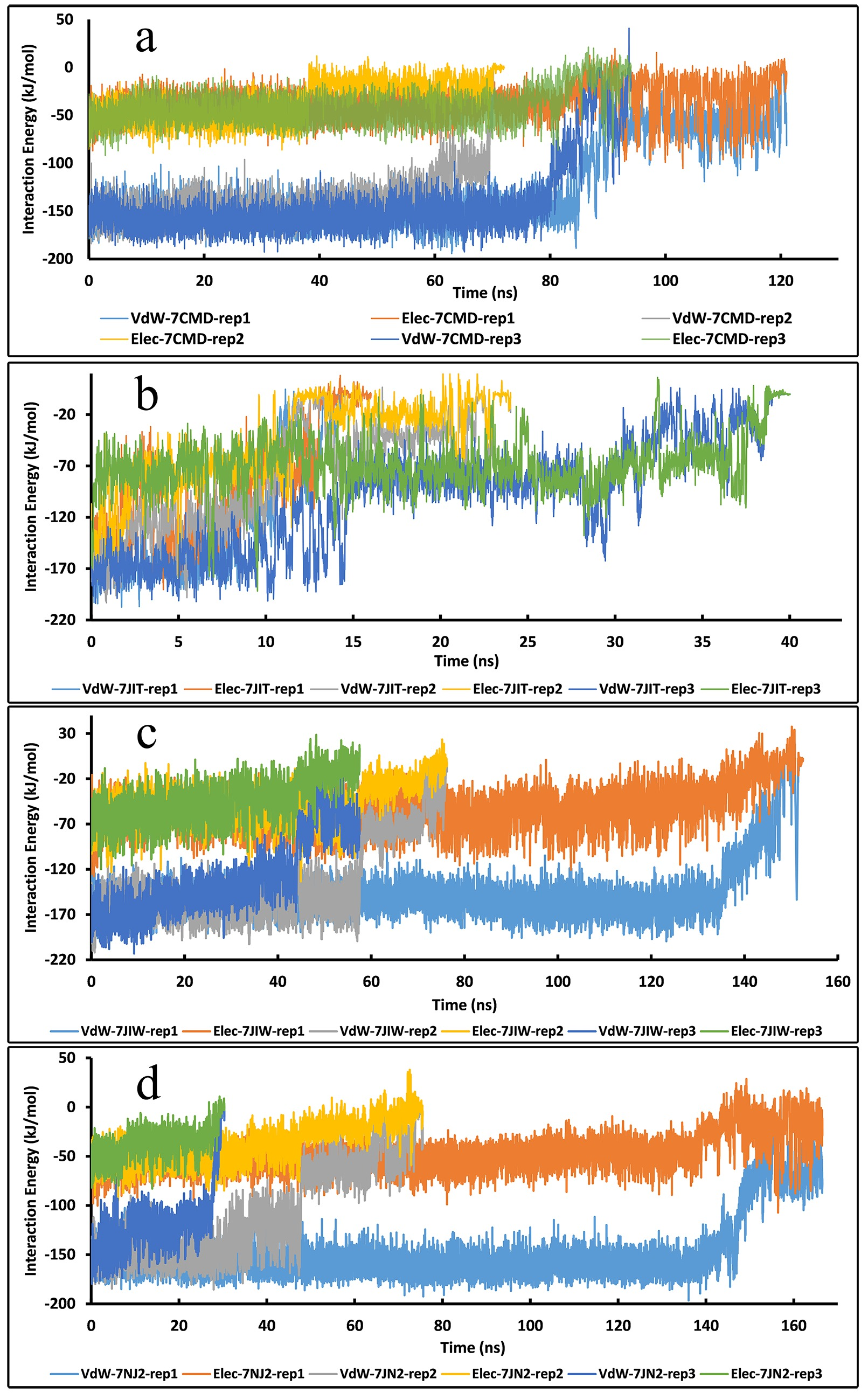

Supplement: S4 Fig — (A) GRL0617-Plpro complex. (B) PLP-Snyder441-Plpro complex. (C) PLP_Snsyder495-Plpro complex. (D) PLP_Snsyder530-Plpro complex. (TIF) [file pone.0251910.s004.tif]

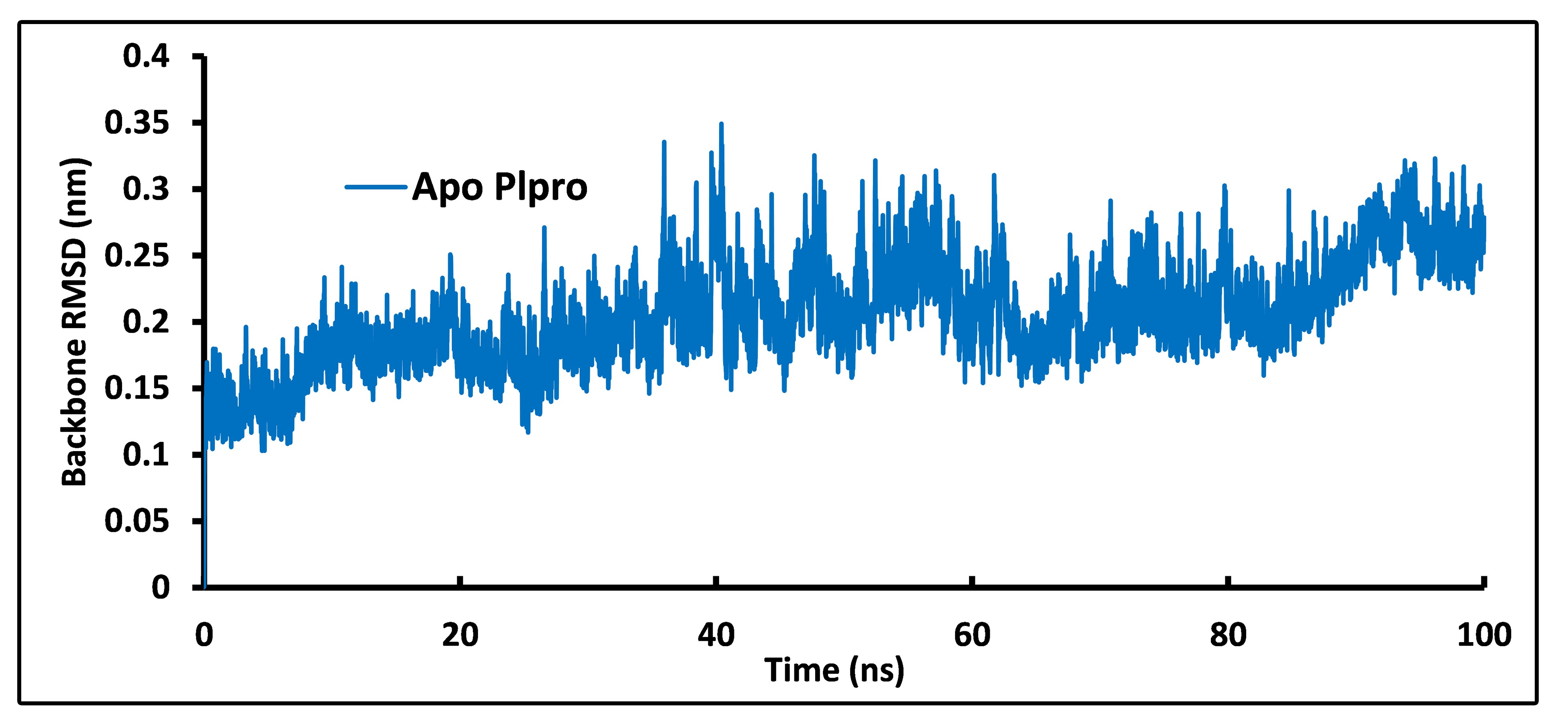

Supplement: S5 Fig — The duration of the simulation was 100 ns. (TIF) [file pone.0251910.s005.tif]
